# “*Candidatus* Colwellia aromaticivorans” sp. nov., “*Candidatus* Halocyntiibacter alkanivorans” sp. nov., and “*Candidatus* Ulvibacter alkanivorans” sp. nov. Genome Sequences

**DOI:** 10.1128/MRA.00086-19

**Published:** 2019-04-11

**Authors:** Mariana E. Campeão, Jean Swings, Bruno Sergio Silva, Koko Otsuki, Fabiano L. Thompson, Cristiane C. Thompson

**Affiliations:** a Laboratory of Microbiology, Instituto de Biologia, Universidade Federal do Rio de Janeiro (UFRJ), Rio de Janeiro, Brazil; b Center of Technology-CT2, SAGE-COPPE, Federal Universidade Federal do Rio de Janeiro (UFRJ), Rio de Janeiro, Brazil; c Ghent University, Ghent, Belgium; Loyola University Chicago

## Abstract

Unplanned oil spills during offshore production are a serious problem for the industry and the marine environment. Here, we present the genome sequence analysis of three novel hydrocarbon-degrading bacteria, namely, “*Candidatus* Colwellia aromaticivorans” sp. nov., “*Candidatus* Halocyntiibacter alkanivorans” sp. nov., and “*Candidatus* Ulvibacter alkanivorans” sp. nov.

## Abstract (precis)

Unplanned oil spills during offshore production are a serious problem for the industry and the marine environment. Here, we present the genome sequence analysis of three novel hydrocarbon-degrading bacteria, namely, “*Candidatus* Colwellia aromaticivorans” sp.

## ANNOUNCEMENT

Metagenomic data obtained from microcosm experiments with oil incubation in seawater from the Foz do Amazonas and Barreirinhas basins indicated the biodegradation potential of deep-sea microbial communities (1). Here, we present the metagenome assembled genome (MAG) sequences of three novel hydrocarbon-degrading bacteria obtained from these previous microcosm experiments, as detailed in our previous work (1). First, read trimming was obtained with prinseq v0.20.4 (-trim_qual_right/trim_qual_left 20, -trim_qual_type min, -trim_qual_rule lt, -trim_qual_window 5, -trim_qual_step 1, -min_len 35, -min_qual_mean 20, -trim_left/-trim_right 10, -out_bad null, -derep 1, and -custom_params\“CTGTCTCTTATACACATCT 1;CTGTCTCTTATACACATCTGACGCTGCCGACGA 1;CTGTCTCTTATACACATCTCCGAGCCCACGAGAC 1\) (2). Subsequently, a binning approach was performed through cross-assembly using eight metagenomes from Foz do Amazonas and eight metagenomes from Barreirinhas with SPAdes v3.6.2 (3); using k-mers of 21, 33, 55, 77, 99, and 127; mapping reads against assembled scaffolds with Bowtie 2 v2.2.5 (4) (–very-fast, -a, –no-unal, –no-discordant, and –no-mixed); converting to bam format with SAMtools v1.7 (5); coverage estimating with jgi_summarize_bam_contig_depths (–outputDepth); and binning with MetaBAT v0.25.4 (–specific, -v, -m 2500, and –minSamples 5) (6). RefineM v0.0.23 (scaffold_stats, outliers, and filter_bins commands) (7) was used to remove contamination, and CheckM v1.0.11 (lineage_wf workflow) (8) was used to assess quality stats. Genomic taxonomy was performed as described previously (9–12). These three novel MAGs showed less than 98.7% 16S rRNA sequence identity with the closest species. The three novel lineages had <95% amino acid identity (AAI) and <70% DNA-DNA hybridization (DDH) toward the closest phylogenetic neighbors, suggesting that the recovered genomes are new species, namely, “*Candidatus* Colwellia aromaticivorans” sp. nov., “*Candidatus* Halocyntiibacter alkanivorans” sp. nov., and “*Candidatus* Ulvibacter alkanivorans” sp. nov. (Table 1). “*Candidatus* Ulvibacter alkanivorans” sp. nov. and “*Candidatus* Halocyntiibacter alkanivorans” sp. nov. possess an alkane monooxygenase, the specific enzyme for alkane degradation, that was lacking in “*Candidatus* Colwellia aromaticivorans” sp. nov. “*Candidatus* Colwellia aromaticivorans” has a phenol 2-monooxygenase gene involved in toluene biodegradation and harbors two monooxygenases and nine oxidoreductases. Oxygenases, a class of oxidoreductases, are key enzymes in hydrocarbon degradation (13).

**TABLE 1:** Genomic features of metagenome-associated genomes^*a*^

Genome feature	Data for strain:		
	“*Candidatus* Colwellia aromaticivorans” sp. nov.	“*Candidatus* Halocynthiibacter alkanivorans” sp. nov.	“*Candidatus* Ulvibacter alkanivorans” sp. nov.
Coverage (×)	97	19	19
Completeness (%)	97.4	98.7	93.1
Contamination (%)	1.8	2.15	0.2
Size (Mbp)	3.71	4.7	2.7
No. of contigs	101	171	117
*N*_50_ value (bp)	72,862	55,069	47,750
No. of ORFs^*b*^	3,278	4,540	2,625
16S rRNA similarity with closest relative (%)	Colwellia rossensis S51-W(gv)1^T^ (98.5)	Halocynthiibacter arcticus PAMC 20958^T^ (97.2)	Ulvibacter antarcticus IMCC3101^T^ (96.2)
GGD^*c*^ with closest relative (%)	*Colwellia rossensis* S51-W(gv)1^T^ (ND)^*d*^	Halocynthiibacter arcticus PAMC 20958^T^ (19.4)	Ulvibacter antarcticus *DSM 23424* (17.8)
AAI with closest relative (%)	Colwellia rossensis S51-W(gv)1^T^ (ND)	Halocynthiibacter arcticus PAMC 20958^T^ (65.4)	Ulvibacter antarcticus DSM 23424 (71.8)

a 16S rRNA sequences recovered were retrieved with RNAmmer (14) and compared with BLAST (15) against the Silva database (16, 17). The closest were selected to align through ssu-align v0.1.1 (18, 19) and perform phylogeny through IQ-TREE (20–22). The 16S rRNA similarity was calculated with TaxonDC (23). AAI and DDH values were performed with CompareM (https://github.com/dparks1134/CompareM) and GGDC (http://ggdc.dsmz.de/), respectively. The genome of Ulvibacter antarcticus DSM 23424 was used for AAI and DDH analysis. *In silico*-predicted phenotype analysis reveals that “*Candidatus* Colwellia aromaticivorans” sp. nov. is positive for chitin hydrolysis, propionate utilization, and dl-lactate utilization. “*Candidatus* Halocynthiibacter alkanivorans” sp. nov. is positive for acid production from d-ribose, sucrose, and glycerol; acetate utilization; indole production; and nitrate reduction. “*Candidatus* Ulvibacter alkanivorans” sp. nov. is positive for dl-lactic acid utilization.

b ORFs, open reading frames.

c GGD, genome-to-genome distance.

d ND, not determined.

### *“Candidatus* Colwellia aromaticivorans” (ar.o.ma’ti.ca. N.L. adj. *aromaticivorans*, aromatic, referring to the property of utilizing aromatic compounds).

*In silico*-predicted phenotypes indicate that the novel species hydrolyzes chitin, utilizes propionate and dl-lactate, and does not utilize d-fructose, l-arabinose, glycerol, citrate, or l-malate. The G+C content is 38%.

### *“Candidatus* Halocyntiibacter alkanivorans” (al.ka.ni’vo.rans. M.L. n. *alkanum* saturated aliphatic hydrocarbon; L. v. *vorare* to eat; L. adj. *alkanivorans* alkane-devouring).

*In silico*-predicted phenotypes indicate that the novel species produces acid from d-ribose, sucrose, and glycerol. Acetate is utilized, nitrate is reduced, and indole is produced. The G+C content is 59%.

### *“Candidatus* Ulvibacter alkanivorans” (al.ka.ni’vo.rans. M.L. n. *alkanum* saturated aliphatic hydrocarbon; L. v. *vorare* to eat; L. adj. *alkanivorans* alkane-devouring).

*In silico*-predicted phenotypes indicate that the novel species utilizes dl-lactate and does not hydrolyze starch or carboxymethyl cellulose (CM-cellulose). It is negative for the utilization of d-mannitol, cellobiose, d-fructose, l-fucose, d-galactose, myo-inositol, d-sorbitol, sucrose, trehalose, and propionic acid. The G+C content is 40%.

### Data availability.

This whole-genome shotgun project has been deposited in GenBank under the BioProject accession no. PRJNA478776.
